# Structure Optimization for Large Gene Networks Based on Greedy Strategy

**DOI:** 10.1155/2018/9674108

**Published:** 2018-06-14

**Authors:** Francisco Gómez-Vela, Domingo S. Rodriguez-Baena, José Luis Vázquez-Noguera

**Affiliations:** ^1^Division of Computer Science, Pablo de Olavide University, 41013 Seville, Spain; ^2^Carrera de Ingeniería Informática, Universidad Americana, Asunción, Paraguay

## Abstract

In the last few years, gene networks have become one of most important tools to model biological processes. Among other utilities, these networks visually show biological relationships between genes. However, due to the large amount of the currently generated genetic data, their size has grown to the point of being unmanageable. To solve this problem, it is possible to use computational approaches, such as heuristics-based methods, to analyze and optimize gene network's structure by pruning irrelevant relationships. In this paper we present a new method, called GeSOp, to optimize large gene network structures. The method is able to perform a considerably prune of the irrelevant relationships comprising the input network. To do so, the method is based on a greedy heuristic to obtain the most relevant subnetwork. The performance of our method was tested by means of two experiments on gene networks obtained from different organisms. The first experiment shows how GeSOp is able not only to carry out a significant reduction in the size of the network, but also to maintain the biological information ratio. In the second experiment, the ability to improve the biological indicators of the network is checked. Hence, the results presented show that GeSOp is a reliable method to optimize and improve the structure of large gene networks.

## 1. Background

One of the most important challenges in systems biology is to understand how individual biological components behave and interact in the context of large and complex systems [1]. This knowledge provides the opportunity of controlling and/or optimizing different parts of biological processes to generate a specific effect in the whole system. Therefore, this system-wide view may lead to new applications in areas such as biotechnology and medicine [2]. In particular, the high amount of data generated in the last years allows the inference of relationships between DNA, RNA, proteins, and other cellular components. The sum of these interactions leads to various types of interaction networks (including protein-protein interaction, metabolic, signalling, and transcription-regulatory networks) called gene networks for the sake of simplicity.

Gene networks are usually inferred from gene expression data and have been widely used to model gene relationships in a biological process [3]. In the last decade, many computational approaches have been proposed for the reverse engineering of gene networks [4]. However, the continuous advances in high-throughput technologies enable carrying out large-scale analyses on the DNA and RNA levels the same as on the protein and metabolite level. As a result, the sources of data from which the gene networks are inferred have increased in size, complexity, and diversity [2]. Due to this, new computational challenges have arisen. For example, some methods have been redesigned to improve their performance during large-scale dataset processing [5]. Other research works have focused their efforts on integrating different sources of data for a more accurate gene network reconstruction, such as the work of [6], in which time data sets from different perturbation experiments are simultaneously considered, or that in [7], where the proposed model integrates big data of diverse types to increase both the power and accuracy of networks inference. Different inference algorithms are combined for reconstructing genome-scale and high-quality gene network from massive-scale RNA-seq samples in [8]. Even other works, like [9], adapt known gene network construction methods to highly parallel execution using distributed high-throughput computing resources.

As a result of these new researches, inferred gene networks are more complex and larger. This fact makes it difficult to visually detect interesting connections between nodes, even though analysis tools have been created recently to apply both advanced statistics and innovative visualization strategies to support efficient knowledge extraction from gene networks [10]. Regarding the gene network structure, some pieces of evidence, like those from the analysis of metabolism and genetic regulatory networks, have proven most biological networks to be sparse, following a scale-free topology. That is, the nodal degree distribution of the network is a power law distribution [11]. Scale-free networks are highly nonuniform; that is, most of the nodes have only a few links while a few nodes have a very large number of links, which are called Hubs. Hubs in a network play a crucial role in how the information is processed in the network since they connect different highly interconnected group of nodes (modules) that could represent different biological functions [12]. Nowadays, the generation of gene networks with a scale-free topology is harder due to the great size and complexity of the networks obtained from the high quantity of data available, so the optimization of gene network structures is currently an important challenge.

In this paper, a new method for automatic optimization of the topology of a large gene network is presented. The method, called Gene Network Structure Optimization (GeSOp), is a backward elimination procedure based on a greedy heuristic method to perform a prune of the irrelevant relationships of the input network. Through this novel method, large genetic networks can improve their topological characteristics without losing their biological information.

### 1.1. Related Works

Explicit structure optimization methods examine networks models and apply a scoring function to assess the degree to which the resulting structure explains the data, while penalizing the complexity of the model. For this aim, interactions are added and/or removed until the best score is reached. Therefore, heuristic search algorithms are one of the most used techniques since exploring all possible combinations of interactions is an NP-hard problem, specially with very big and complex networks [2, 13]. Several optimization techniques have been developed. However, they are usually limited by the high dimensionality of the problem, as well as computational power required for large networks [14].

Some research works use evolutionary techniques. To reduce the large search spaces, elitist selection method is often used in genetic algorithms, ensuring that the algorithm does not waste time in the rediscovery of previously discarded partial solutions. For example, in [15], a random Boolean network is evolved to look for an accurate model based only on experimental data, without taking into account prior biological knowledge. Other research works use other methods to improve the algorithm's performance, like [16] that proposes a multiagent genetic algorithm to reconstruct large-scale gene regulatory networks. This algorithm is based on fuzzy cognitive maps and includes efficient search operators to reduce the search space.

The optimization algorithms that are based on one objective function, for example, error minimization, can lead to over-fitting and many false positive connections in large networks inference. For example, in [17], the inference problem of *N* genes is decomposed into *N* × (*N* − 1) different regression problems, in which the expression level of a target gene is predicted from the expression level of a potential regulation gene by using the sum of squared residuals and the Pearson correlation coefficient. To reduce the over-fitting phenomena, some works use multiple objective functions and/or add prior biological knowledge to infer an accurate network model. For example, authors in [18] import some a priori regulatory information about extracted gene networks from existing publications or biological web sites with the aim of enhancing veracity of the network. The proposal presented in [19] was the first one to incorporate functional association databases. They create undirected, confidence-weighted likelihood matrix by means of pairwise confidence scores from those databases and use it to infer gene networks, improving their accuracy.

Other works focus their efforts on looking for scale-free properties. For example, in [20], a new proposal is presented which takes the scale-free topology into account as prior information to prune the search space during the inference process. This way, the search space traversed by the method integrates the exploration of all predictors sets combinations, like when having a small number of combinations, when performing a floating search, or when the number of combinations becomes excessive.

This process is guided by scale-free prior information. In [21], informative prior based on scale-free property is also used to improve inference accuracy. In particular, during a Bayesian-based inference process, prior knowledge about scale-free properties is used to evaluate the relative importance of nodes from the linkage characteristics of the entire network.

As can be observed, most research works in literature integrate different network structure optimization strategies within the inference process. Therefore, these optimization efforts depend on concrete input data and the network generation tasks. In this sense, to the best of our knowledge, the new method proposed in this paper is the first one that is independent of the network inference process. As a result, this method is able to optimize any input gene network.

## 2. Materials and Methods

In this section, the methods and the different materials used in this paper are presented. Firstly, the GeSOp method to optimize large gene network structures is exhaustively described. Secondly, the gene network generation method applied in the experimentation will be presented, along with the input datasets and biological databases used.

### 2.1. Gene Network Structure Optimization

GeSOp is a novel method for large gene networks topology optimization. The method uses undirected influence networks since they represent the highest level of abstraction in the gene networks as was discussed in [3]. Due to this, our method can be applied for a larger number of networks since almost any gene network can be transformed into a nondirected influence network.

**Figure 1:**
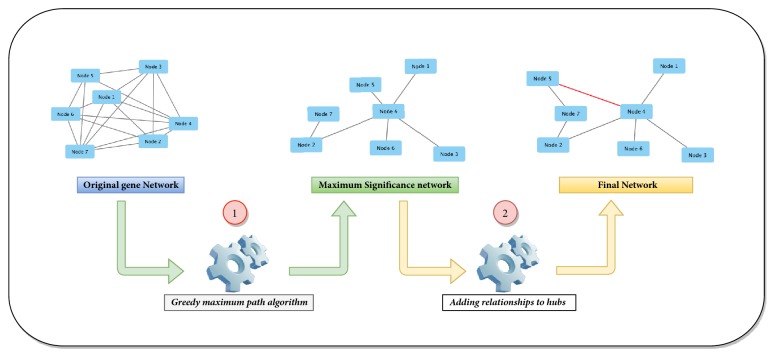
GeSOp method is composed of two different steps: 1. application of a greedy algorithm to prune the original network and 2. detection of Hubs in the resulting network and their enrichment by adding new interactions.

The main goal of the GeSOp is to transform the input gene network into a simpler and more efficient network in terms of information transfer, keeping the biological meaningfulness [2]. For this aim, a new backward removal procedure composed of two different steps has been developed. Initially, GeSOp uses a greedy-based heuristic strategy to prune the original network and select the most biologically relevant interactions. Then, the method looks for the most connected nodes (Hubs) in the resultant network and proceeds by adding relevant interactions which were pruned on the previous step. A description of the general schema of the method, along with a toy example, is shown in Figure 1. A complete description of the two steps and a pseudocode of the method are detailed below.

**Figure 2:**
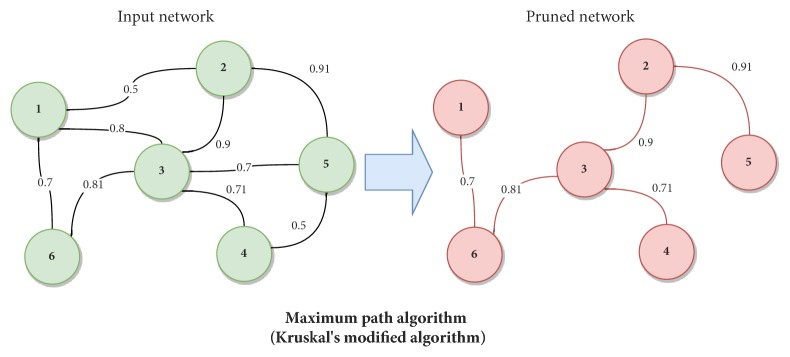
Representation of step 1, in which an input network is pruned using the maximum path algorithm.

*Step1: Greedy Maximum Relevance Path.* The first step of GeSOp uses a greedy-based heuristic algorithm to perform a prune of the input network, taking into account most relevant interactions from a biological point of view (see Figure 2). To do so, a modification of Kruskal's algorithm for the shortest path problem in graphs has been developed [22].

In particular, our method does not select the shortest path between nodes. On the contrary, it selects the longest path according to the weight of edges. Therefore, the relationships with the highest level of significance are selected with respect to the weight of the edges for later network reconstruction.

As a result, the pruned network generated contains the same number of genes (nodes) as the original network but it keeps only most relevant relationships. Hence, it implies a large reduction in terms of the number of edges, while still depending on the degree of connectivity of the original network, as is shown in Figure 2.

**Figure 3:**
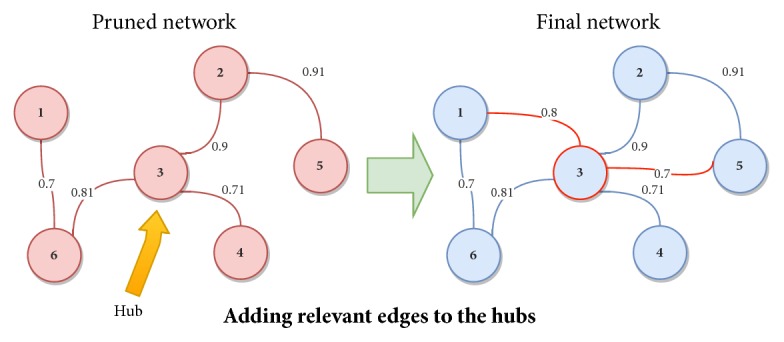
An example of the second step of our method, in which the Hubs of the pruned network are identified and relevant edges are added to them. Note that the relationships are added if their weight exceeds the *Th*_*β*_; in this example, *Th*_*β*_ ≥ 0.7.

*Step2: Addition of Missing Relationships.* As is mentioned in Section 1, Hubs have been reported to have special properties regarding their neighbouring nodes in a gene network. Due to this, in this second step, a topological analysis of the pruned network is performed in order to identify network's Hubs. For this aim, Hubs are selected as those nodes whose connection degree exceeds average network connectivity [12]. A toy example is depicted in Figure 3, where the node “3” is identified as a Hub on the left network.

After the Hubs identification, a threshold (*Th*_*β*_) is set to determine which relationships of those removed in step 1 should be added to the Hubs. The threshold *Th*_*β*_ is an input parameter of GeSOp algorithm (see Algorithm 1) and it is determined by the user. In this sense, the user may select the threshold which better fits the problem studied. Thus, a new relationship is added to the final network if exceeding *Th*_*β*_. The process is represented in Figure 3, where two pruned relationships are added to the Hub node in the network on the right.

The final network is generated after each Hub of the pruned networks is processed.

A general pseudocode of the complete method described in this paper is presented in Algorithm 1.

Finally, the complexity of GeSOp combines the complexity of the Step1 (Θ(*E*log⁡(*V*))) and the Step2 (Θ(*V*(*E*^2^))) resulting in and average case complexity of

(1) $$\begin{array}{c} \Theta \left(\;E\log\left(\;V\;\right)\;\right)+\Theta \left(\;V\left(\;E^{2}\;\right)\;\right), \end{array}$$

where V and E represent the number of genes and relationships of the input network, respectively.

### 2.2. Input Datasets

In this section, experimental datasets used for the generation of input gene network used to test GeSOp implementation are shown. In particular, we have selected two different datasets from two different organisms with different features.

*Saccharomyces cerevisiae Cell Cycle Dataset.* The first dataset used was the one presented by Spellman et al. [23], in relation to the well-known Yeast Cell Cycle. This microarray describes the expression level of 5521 genes in samples from yeast cultures, which were synchronized by three independent methods: *α* factor arrest, elutriation, and arrest of a cdc15 temperature-sensitive mutant. Particularly, we focus on data generated by cdc15 experiments.

*Homo sapiens Single Nucleotide Polymorphism (SNP) Dataset.* In order to prove the usefulness of our proposed method, the* Homo sapiens* SNP, presented in the work of Hodo et al. [24], has been also selected. This dataset was obtained to study associations of interleukin 28B with carcinoma recurrence in patients with chronic hepatitis C, and it contains information about 54616 genes of* Homo sapiens*.

### 2.3. Gene Networks Generation Methods

In the following, the methods used to extract gene networks from the two datasets presented above are described. In total, three networks were generated for each dataset. Gene networks based on information theory are one of the most widely used types in literature [2] since they are able to identify coexpression relationships among genes. In this sense, we have selected this kind of networks since they are computationally simple and allow the fitting of large datasets. In particular, three standard measures from information theory to generate coexpression gene networks have been used:** Spearman's** correlation algorithm,** Kendall's** Rank correlation algorithm [1, 25], and** Symmetric Uncertainty** measure (SU) [26, 27].

**Figure 4:**
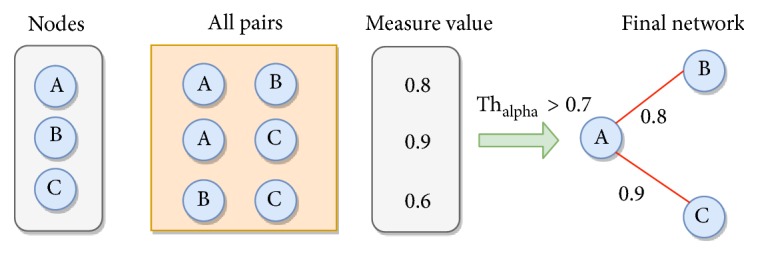
An example of the generation of the input networks. Note that the relationships are added if their weight exceeds the *Th*_*α*_.

Gene networks were constructed by calculation of the presented measures (Kendall, Spearman, and SU) from the expression levels in each pair of genes from the input datasets. If the result of the measure exceeds a determinate threshold (here after *Th*_*α*_) selected by the user, a new edge is added to the network between the nodes as is represented by Figure 4.

For our study, we have selected a low threshold, *Th*_*α*_ = 0.5, in order to obtain over-connected networks as was discussed in [3].

### 2.4. Biological Databases

The aim of this section is to present the biological databases used as reference in the experiment section.

In particular, we have selected three different databases: (a) the GeneMANIA database for evaluating yeast and human networks, (b) YeastNet database for yeast, and (c) HumanNet for human.

**GeneMANIA** [28] contains information presented in the form of web application for generating hypotheses about gene functions. A prediction server uses a large set of functional association data, including protein and genetic interactions, pathways, coexpression, colocalization, and protein domain similarities. The information stored in GeneMANIA is freely available online. This information is stored in a structure categorized by organisms, where genes (nodes) are related (gene-gene relationship) if at least one piece of evidence of this relation exists in the literature.

**YeastNet**, which was presented in [29], is a probabilistic functional gene network obtained from 5794 protein-coding genes of the yeast extracted from* Saccharomyces cerevisiae* Genome Database [30]. This network combines protein-protein interactions, protein-DNA interactions, coexpression, phylogenetic conservation, and also literature information, in total covering 102803 linkages among 5483 yeast proteins.

Finally** HumanNet**, which was presented in [31], is a probabilistic functional gene network of 18714 validated protein-coding genes of* Homo sapiens*. It is constructed by modified Bayesian integration of 21 types of “omics” data from multiple organisms. Each data type is weighted according to how well it associates known genes to a biological function in* Homo sapiens*. Each interaction in HumanNet has an associated log-likelihood score that rates the probability of a relationship representing a true functional linkage between two genes.

## 3. Results and Discussion

The performance of the proposed method was tested by means of two different experiments. The aim of the first experiment is proving that the networks processed by our method do not lose rate of biological information. To this end, we have used different networks, generated using standard methods of literature, and different databases (see Sections 2.3 and 2.4). In the second experiment, a topological analysis of different networks is carried out to check how biological structure indicators are improved.

### 3.1. Biological Information Analysis

The aim of this experiment is to show how the networks processed by our method reduce the size of the network, keeping their biological information ratio. To do so, for each dataset used, we present a comparison, in terms of size and performance, between the original inferred network and those optimized by GeSOp.

#### 3.1.1. Performance Evaluation

The quality of the optimized networks was assessed by a direct comparison with a gold standard, that is, the biological databases presented in Section 2.2. To compute the quality measures, the following indices were defined as they were presented in [32]:

- **True positives (TP):** both networks contain the gene-gene relationship evaluated.
- **False positives (FP):** the input network contains a relationship which is not present in the biological database.
- **True negatives (TN):** the relationships are not present neither in the input network nor in the biological database.
- **False negatives (FN):** the relationship exists in the biological database but it does not in the input network.

Once these indices are obtained, other measures used in the literature have been selected to rate the quality of gene networks [2, 3],* Precision* and* Recall* [2, 33], which are defined below.

(2) Precision=TPTP+FP

(3) Recall=TPTP+FN

#### 3.1.2. Yeast Experiment

As was stated before, in this subsection, the results obtained by the networks generated by the Yeast Cell Cycle dataset are presented. The input networks were generated using a *Th*_*α*_ = 0.5 as cut-off to generate over-connected networks as was introduced in [3]. On the other hand, GeSOp uses a threshold *Th*_*β*_ = 0.7 for adding relationships. We have selected this threshold as relevant correlation value as was also discussed in [3].

The first analysis is presented in Table 1, in which the number of nodes and edges of the original networks and the optimized ones are exposed.

The table presents the different results obtained by the networks generated by the following methods: Kendall, Spearman, and SU. The first column of each method represents the original input network (network obtained by method on the dataset with *T*_*h*_ = 0.5) and the second one (“GeSOp”) the final network obtained by our method. On the other hand, the rows of the table represent the number of nodes presented in the network (“Nodes”) and the number of relationships comprising the network (“edges”), respectively. Finally, the column “diff. %” represents the difference between the number of edges of the input and final network.

Firstly, it is worth mentioning that the network generation methods present different results for the same dataset. Spearman's method is the one that obtains larger networks since the method is able to find less strictness coexpression levels. On the other hand, SU's method is the most restrictive, as this technique is based on detecting not only the lineal dependencies, but also the nonlinear ones. Finally, Kendall's method is more restrictive than the Spearman method but more relaxed than the SU's.

Regarding the size of the networks, results show that the networks optimized by GeSOp have reduced their size from 81,81% to 98.25%, in terms of number of edges. Note that GeSOp preserves the nodes, as was described previously. These results represent a significant size reduction, which implies that the final networks are simpler and more user-friendly for researchers in terms of size and visualization.

Once it has been shown that GeSOp is capable of carrying out a reduction in the size of gene networks, it is also important to check if these optimized networks keep the ratio of biological information that they originally contained. For this aim, Tables 2 and 3 are presented. In them, for each method of generation (i.e., Kendall, Spearman, and SU), three columns are displayed. The columns “Input” represent the results for the input network, columns “GeSOp” represent the optimized networks generated by GeSOp. In addition, the results obtained by the networks computed only in step 1 of our method are presented in the “Pruned” columns. The rows “Precision” and “Recall” indicates the ratio of biological information of the networks according to the biological databases used.

Results show that the networks do not suffer any loss of information. On the contrary, the value of the Precision measure for these networks is increased. For example, in the case of the Kendall's network compared to YeastNet, Precision value goes from 0.01 to 0.09, which is a significant improvement. This behaviour is also presented in the Spearman's and SU's networks, where Precision's values increase from 0.01 to 0.02.

Regarding the Recall, it has been reduced in all the networks optimized by our method. This fact makes sense, since Recall value is inversely proportional to the number of FN, which are the relationships that are present in the biological databases. Therefore, our method for reducing the size of the network is inherently increasing the number of FN. Thus, the greater the database used to rate the network, the lower the value of its Recall because there will be more FN.

#### 3.1.3. *Homo sapiens* Experiment

In this subsection, the experiments carried out by means of the human SNP dataset are described. The obtained networks were generated using the same parameters as in the previous section (*Th*_*α*_ = 0.5 and *Th*_*β*_ = 0.7).

The analysis carried out on the size of the different human networks is shown in Table 4. The results follow the same pattern as of the yeast networks. Spearman is the method which presents the larger network while SU presents the smaller.

GeSOp is able to reduce considerably the size of the networks (e.g., −85.68% for Kendall's network and −89.46% for Spearman's), but the case of SU's network is remarkable. In this case, the reduction is about −40.08%, which is significantly lower than the rest of the cases. This result is consistent with the fact that the SU's network is significantly smaller than the rest of the studied networks, so it is difficult to reduce the size of this network without losing biologically relevant relationships. Due to this result, it is possible to argue that GeSOp performs better with larger gene networks which contain spurious relationships.

The biological validation of the different networks using GeneMANIA and HumanNet databases (see Section 2.2 for more details) is presented in Tables 5 and 6, respectively.

The validation results follow the same pattern as for the yeast networks. The accuracy value increases for all cases except for SU's networks. As was discussed above, it is difficult to prune small networks without losing relevant relationships. Even so, the loss of Precision value is very small (0.04 with GeneMANIA and 0.01 on HumanNet).

In conclusion, the results obtained by both experiments show how GeSOp is able to perform a pruning process on large networks, by reducing their size while keeping their ratio of biological information. The relevance of our method became more evident since, as was discussed in literature [14], the optimization usually implies loss of information in the majority of the cases. However, for almost all analyzed cases, Precision of the network is improved by GeSOp.

### 3.2. Topological Analysis

In this section, the ability of GeSOp to improve the topology of gene networks is analyzed.

As was stated in Section 1, biological networks usually follow topological patterns, in particular the scale-free topology. The topology of a network is crucial to understand the biological network's architecture and performance [34]. Therefore, gene networks inferred by computational methods should present this type of topology [3]. Based on this assumption, we present a topological analysis of some of the networks optimized by GeSOp in the previous section. The objective is to identify if their topology indicators have been improved in terms of scale-free topology.

Scale-free networks have a structure containing only a few Hubs, among some other features. The most important and commonly used topological features of scale-free networks are presented [35, 36] as follows:

- **Characteristic path length (CPL):** The CPL of a network indicates the shortest path length between two nodes, averaged over all pairs of nodes comprising the network. A high path length indicates that the network is in a linear chain. A lower value means that is more compact. Scale-free networks usually have a great CPL.
- **Diameter**: The diameter of a network indicates the maximal distance between two nodes. As in the case of CPL, a greater diameter of the network indicates that it follows a biological pattern.
- **Clustering coefficient**: For one node, this coefficient can be calculated as the number of links among the nodes within its neighbourhood divided by the number of links that are possible among them. A high clustering coefficient for a network is another indicator of the existence of biological relationships.
- **Graph density:** The density of a network defines the ratio of the number of edges to the number of possible edges. Gene networks are generally sparsely connected. Therefore, a low density should indicate biological meaning in the network.
- **The node degree distribution**: It indicates the ratio of nodes in the network with degree *k*. Scale-free networks usually follow a power law: *P*(*k*) ~ *k*^*γ*^, where *γ* is a constant (≥0). A high *γ* is an indicator of a scale-free topology.

**Figure 5:**
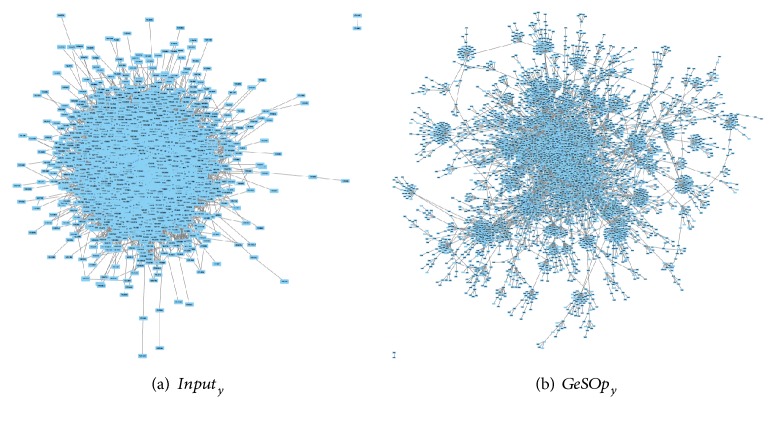
Visual comparison of yeast network. The original Kendall's network is shown on (a). On (b), the final network obtained with GeSOp is depicted. As can be observed, the optimized network presents a scale-free topology.

**Figure 6:**
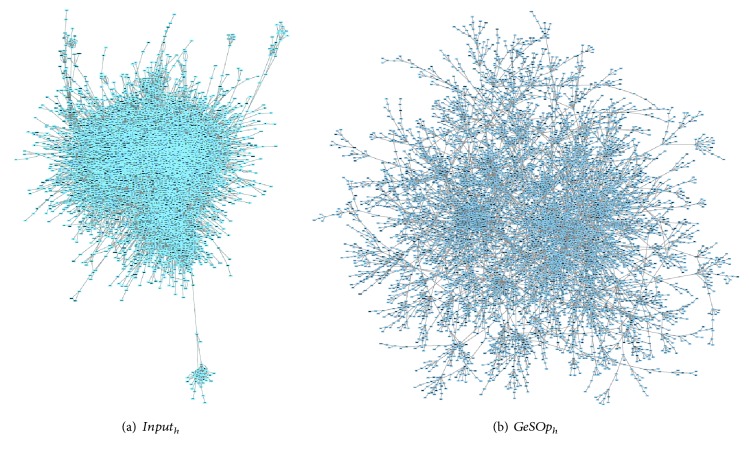
Visual comparison of human networks used in this experiment. The original Kendall's network is shown on (a). On (b), the optimized network obtained with GeSOp is depicted.

For this experiment, the networks obtained by Kendall's method on Yeast and Human datasets have been used as reference, for the sake of simplicity. Thus, we present a topological study for four networks, the originals (named “*Input*_*organism*_”) and the processed ones (hereafter “*GeSOp*_*organism*_”). Visual representation of the networks is depicted in Figures 5 and 6, where it is possible to check the topological differences of the networks.

As can be seen in the figures, the optimized networks (“*GeSOp*_*x*_”) present a more linear and less compact topology than the input ones, so they fit better with the scale-free topology. In addition, an exhaustive topological analysis of the four networks has been carried out based on the indicators presented above. The topological analysis of the network has been performed using the tool Network Analyzer [37] and the results obtained are depicted in Table 7.

The results presented in Table 7 show that the networks improve their topological indicators once they are processed by GeSOp. Moreover, it is possible to argue that these networks follow a biological pattern according to [36]. That is, after the optimization process, networks show, on the one hand, a lower mean clustering coefficient and density. On the other hand, they present higher characteristic path length, diameter, and *γ* constant. These results mean that networks have improved in terms of the biological relevance of their relationships.

**Figure 7:**
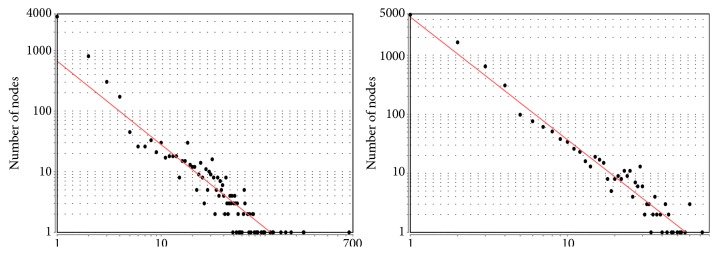
Node degree for the optimized networks obtained with GeSOp. The fitted power law indicates that the networks follow a scale-free topology.

Moreover, the optimized networks present characteristics closer to a scale-free topology as their node degree distribution follows a power law with *γ* ≥ 0[34] (see Figure 7 ). This fact can be verified by the results presented in column “Gamma” of Table 7, in which the values of *γ* (from power law) are improved in the optimized networks.

The results generated by this second experiment probes that GeSOp is a reliable method to improve the topological features of the gene networks, in terms of biological structure.

## 4. Conclusions

In this work, a new backward elimination method for optimization of large gene networks structure, namely, GeSOp, has been presented. The method, which is based on a greedy strategy, is able to perform a drastic reduction of size of the input network in terms of the number of gene-gene relationships. The prune of the less biologically significant relationships produces simpler and more user-friendly networks for researchers in terms of size and visualization.

On one hand, the results presented show that the method is able not only to perform a prune of the input network, but also to keep the ratio of the biological information presented in the original network. Furthermore, for almost all studied cases, this ratio is improved. On the other hand, topological analyses carried out in the experiments show how networks optimized by GeSOp improve their biological indicators by acquiring a scale-free topology. Finally, regarding the generated results, it is possible to argue that the relevance of our method becomes evident for the processing and optimization of large gene networks.

As future works, we will work on the inclusion of previous biological knowledge, in form of gene networks as gold standard, in the second step of the methodology. Thus, the method will take into account not only the existing Hubs in the input network, but also the genes that have a great relevance in the networks used as gold standard. Another future work is based on the implementation; we are working in paralleling implementation of the algorithm to improve its performance.

## Figures and Tables

**Algorithm 1:**
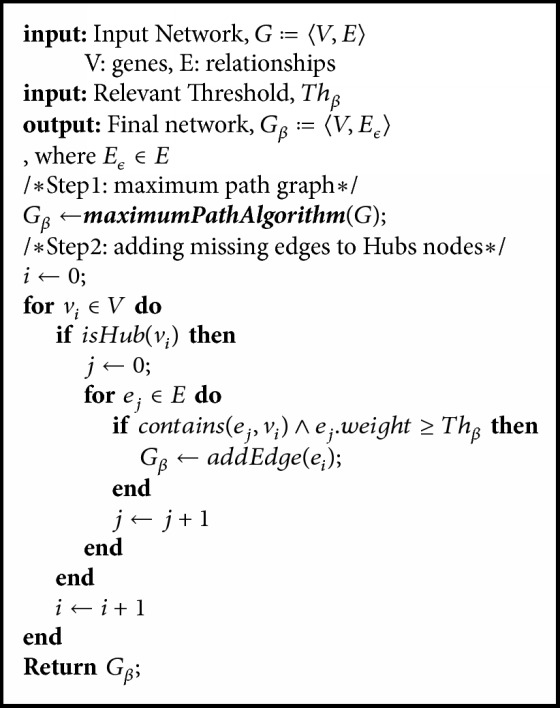
A general pseudocode of the proposed method. The algorithm is divided into two different steps.

**Table 1:** Results of yeast cell cycle networks processed with GeSOp. As it is shown, networks are significantly reduced in size.

**Yeast**									
	**Kendall**			**Spearman**			**SU**		

	**Input**	**GeSOp**	**diff. **%	**Input**	**GeSOp**	**diff. **%	**Input**	**GeSOp**	**diff. **%

**Nodes**	5466	5466	-	5521	5521	-	4802	4802	-

**Edges**	619552	10801	-98.25 %	2555009	446704	-82.51%	145329	26421	-81.81%

**Table 2:** Yeast's network results against YeastNet.

	**Kendall**			**Spearman**			**SU**		
	Input	Pruned	GeSOp	Input	Pruned	GeSOp	Input	Pruned	GeSOp
TP	8331	94	909	19706	64	6589	1744	94	436
FP	444362	4035	9449	1864316	4374	328473	102890	3496	20850
Precision	0.01	0.02	0.094	0.01	0.01	0.02	0.01	0.026	0.02
Recall	0.08	9.18 · 10^−4^	0.009	0.2	6.25 · 10^−4^	0.006	0.01	9.18 · 10^−4^	0.004

**Table 3:** Yeast's network results against GeneMANIA.

	**Kendall**			**Spearman**			**SU**		
	Input	Pruned	GeSOp	Input	Pruned	GeSOp	Input	Pruned	GeSOp
TP	194918	1942	7863	692753	1909	147360	43991	1722	10281
FP	400383	3273	8423	1770378	3326	293279	95244	2824	18206
Precision	0.32	0.37	0.48	0.28	0.36	0.33	0.31	0.37	0.36
Recall	0.04	4.01 · 10^−4^	0.016	0.08	3.94 · 10^−4^	0.003	0.009	3.56 · 10^−4^	0.002

**Table 4:** Results of human SNP networks processed with GeSOp. The size of the networks is also significantly reduced.

**Human**									
	**Kendall**			**Spearman**			**SU**		

	**Input**	**GeSOp**	**diff. **%	**Input**	**GeSOp**	**diff. **%	**Input**	**GeSOp**	**diff. **%

**Nodes**	8068	8068	-	31061	31061	-	1431	1431	-

**Edges**	68329	9783	-85.68%	5387473	567590	-89.46%	1871	1121	-40.08%

**Table 5:** Human's network results against GeneMANIA.

	**Kendall**			**Spearman**			**SU**		
	Input	Pruned	GeSOp	Input	Pruned	GeSOp	Input	Pruned	GeSOp
TP	17144	1282	2085	351686	1305	52563	525	299	303
FP	26416	2759	3116	2512234	11646	248969	745	545	553
Precision	0.39	0.31	0.4	0.12	0.10	0.18	0.40	0.35	0.36
Recall	0.0024	1.83 · 10^−4^	2.98 · 10^−4^	0.04	1.86 · 10^−4^	0.0075	0.7 · 10^−4^	0.4 · 10^−4^	0.43 · 10^−4^

**Table 6:** Human's network results against HumanNet.

	**Kendall**			**Spearman**			**SU**		
	Input	Pruned	GeSOp	Input	Pruned	GeSOp	Input	Pruned	GeSOp
TP	4216	276	586	46850	141	8202	125	77	77
FP	35931	3291	4084	2465035	10540	258413	1045	699	711
Precision	0.10	0.07	0.12	0.01	0.01	0.03	0.10	0.09	0.09
Recall	0.008	5.79 · 10^−4^	0.001	0.09	2.95 · 10^−4^	0.017	2.4 · 10^−4^	1.61 · 10^−4^	1.66 · 10^−4^

**Table 7:** Topological indicator of four selected networks. The results presented show how the optimized networks obtained by GeSOp improve their indicators.

	**Network**	**Clust. coef.**	**CPL**	**Diameter**	**Density**	**Gamma (** *γ * **)**
**Yeast**	*Input*_*y*_	0.411	2.697	9	0.041	0.845
	*GeSOp*_*y*_	0.085	6.156	20	0.001	1.375

**Human**	*Input*_*h*_	0.21	4.954	19	0.003	1.394
	*GeSOp*_*h*_	0.024	10.84	33	~ 0.000	2.079

## Data Availability

In this section, we provide the links to the datasets and databases presented above. In particular, the links for the datasets are as follows: **Yeast dataset**: https://www.ncbi.nlm.nih.gov/geo/query/acc.cgi?acc=GSE23 **Human dataset**: https://www.ncbi.nlm.nih.gov/geo/query/acc.cgi?acc=GPL570 and those for the databases are as follows: **GeneMANIA**: http://genemania.org/data/ **YeastNet**: https://www.inetbio.org/yeastnet/ **HumanNet**: http://www.functionalnet.org/humannet/
